# (9*H*-Fluoren-9-yl)(phen­yl)phosphinic acid

**DOI:** 10.1107/S160053681204891X

**Published:** 2012-12-05

**Authors:** Robert A. Burrow, Rubia M. Siqueira da Silva

**Affiliations:** a Laboratório de Materiais Inorgânicos, Universidade Federal de Santa Maria, 97105–900 Santa Maria–RS, Brazil

## Abstract

The crystal structure of the title compound, C_19_H_15_O_2_P, features pairs of mol­ecules joined by O—H⋯O hydrogen bonds across crystallographic inversion centers. In addition, π–π inter­actions, with a centroid–centroid distance of 3.6273 (9) Å between the fluorene ring systems, connect the dimers into chains along [01-1]. The three rings make dihedral angles of 1.34 (9), 1.52 (10) and 1.51 (7)° with each other.

## Related literature

For related structues, see: Burrow *et al.* (2000 ▶); Vioux *et al.* (2004 ▶); Siqueira *et al.* (2006 ▶); Burrow & Siqueira da Silva (2011*a* ▶,*b* ▶); Burrow & Siqueira da Silva (2012 ▶). For a description of the Cambridge Structural Database and geometry checks using *Mogul*, see: Allen (2002 ▶); Bruno *et al.* (2004 ▶). For hydrogen-bond information, see: Jeffrey (1997 ▶). For the synthesis, see: Boyd & Regan (1994 ▶).
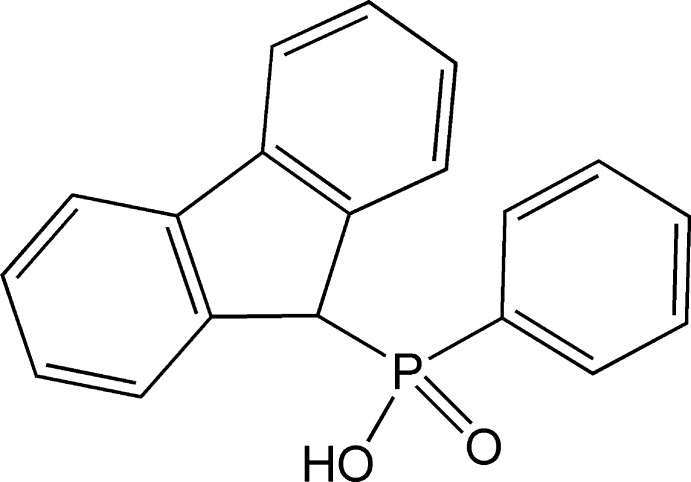


## Experimental

### 

#### Crystal data

C_19_H_15_O_2_P
*M* *_r_* = 306.28
Triclinic, 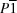

*a* = 8.5736 (7) Å
*b* = 9.5668 (8) Å
*c* = 9.6750 (7) Å
α = 73.348 (5)°
β = 87.388 (5)°
γ = 83.619 (6)°
*V* = 755.49 (10) Å^3^
*Z* = 2
Mo *K*α radiation
μ = 0.19 mm^−1^
*T* = 100 K
0.28 × 0.13 × 0.07 mm

#### Data collection

Bruker X8 Kappa APEXII diffractometer
Absorption correction: numerical (*SADABS*; Bruker, 2012 ▶) *T*_min_ = 0.941, *T*_max_ = 0.988
21221 measured reflections
4614 independent reflections
3560 reflections with *I* > 2σ(*I*)
*R*_int_ = 0.047

#### Refinement

*R*[*F* ^2^ > 2σ(*F* ^2^)] = 0.044
*wR*(*F* ^2^) = 0.112
*S* = 1.03
4614 reflections
202 parameters
H atoms treated by a mixture of independent and constrained refinement
Δρ_max_ = 0.33 e Å^−3^
Δρ_min_ = −0.41 e Å^−3^

### 

Data collection: *APEX2* (Bruker, 2012 ▶); cell refinement: *SAINT* (Bruker, 2012 ▶); data reduction: *SAINT*; program(s) used to solve structure: *SHELXS97* (Sheldrick, 2008 ▶); program(s) used to refine structure: *SHELXL97* (Sheldrick, 2008 ▶); molecular graphics: *DIAMOND* (Brandenburg, 2012 ▶); software used to prepare material for publication: *PLATON* (Spek, 2009 ▶) and *publCIF* (Westrip, 2010 ▶).

## Supplementary Material

Crystal structure: contains datablock(s) global, I. DOI: 10.1107/S160053681204891X/lh5563sup1.cif

Structure factors: contains datablock(s) I. DOI: 10.1107/S160053681204891X/lh5563Isup2.hkl

Supplementary material file. DOI: 10.1107/S160053681204891X/lh5563Isup3.cdx

Supplementary material file. DOI: 10.1107/S160053681204891X/lh5563Isup4.cml

Additional supplementary materials:  crystallographic information; 3D view; checkCIF report

## Figures and Tables

**Table 1:** Hydrogen-bond geometry (Å, °)

*D*—H⋯*A*	*D*—H	H⋯*A*	*D*⋯*A*	*D*—H⋯*A*
O1—H1⋯O2^i^	0.90 (2)	1.61 (2)	2.5107 (15)	177.9 (19)

Symmetry code: (i) 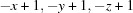.

**Fig. 1.**
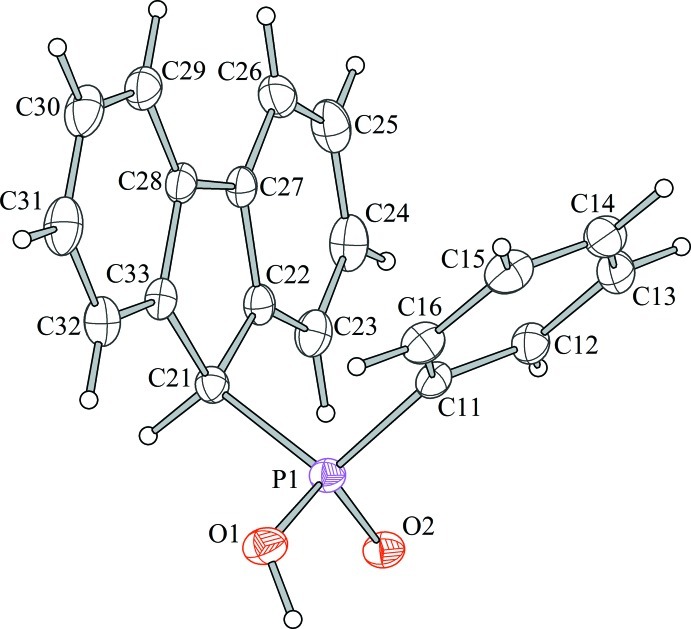
The molecular structure of the title compound, with 50% probability ellipsoids.

**Fig. 2.**
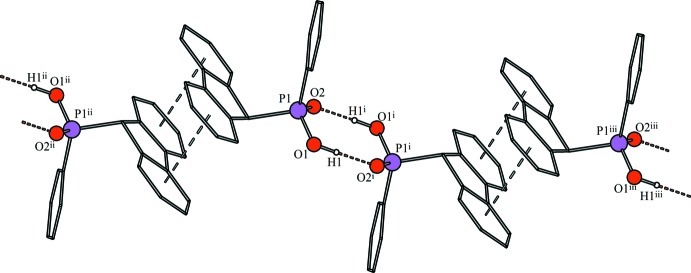
The H bonding interactions, dashed red lines, and π-π interactions, dashed gray lines, which join the molecules of the title compound into continuous chains along the crystallographic [011] direction. Non-essential H atoms are omitted and C atoms are shown as sticks for clarity. Symmetry codes: (i) 1–x, 1–y, 1–z; (ii) 1–x, 2–y, 1–z; (iii) x, -1 + y, 1 + z.

**Fig. 3.**
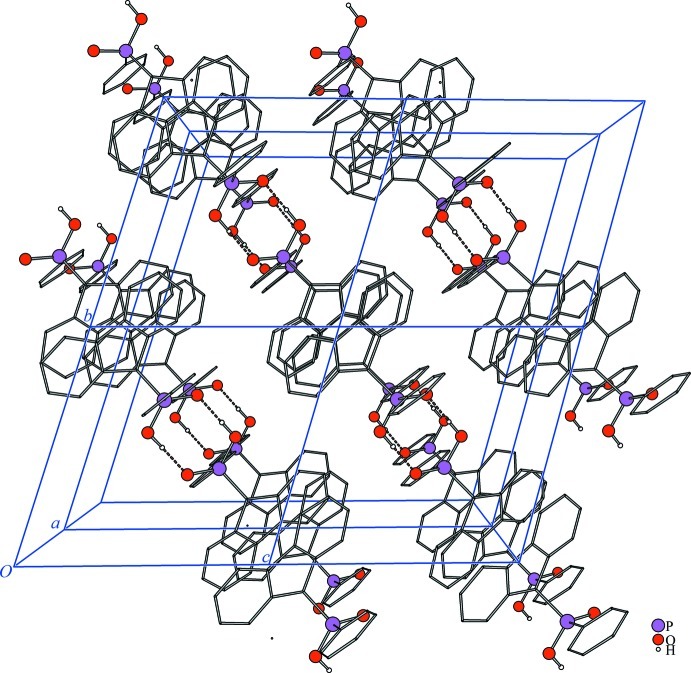
The packing diagram of the title compound in the crystallographic bc direction with the crystallographic a axis pointing down. Non-essential H atoms are omitted and C atoms are shown as sticks for clarity.

## supplementary crystallographic information

### Comment

Phosphinic acids usually form continuous chain structures *via* hydrogen bonding interactions between neighboring phosphinic acid groups in the solid state (Burrow *et al.*, 2000; Burrow & Siqueira da Silva, 2011*a*,*b*; Burrow & Siqueira da Silva, 2012). This tendency, due to the strong P—O dipole moment, aids in the formation of chain like structures in coordination polymers (Vioux *et al.*, 2004; Siqueira *et al.*, 2006). Smaller organyl groups, such as the methyl group, bound to the P atom can direct the the structure to form lamellar structures (Burrow & Siqueira da Silva, 2011*b*). Larger groups such as fluorenyl in the title compound have the ability to do the opposite, reducing the structure to dimeric structures by making chain formation an unfavorable process due to steric interactions between the bulky groups. Here we report the synthesis and crystal structure of the title compound which demonstrates the steric effects of the organyl groups in the solid state.

The molecular structure of the title compound is shown in Fig. 1. The geometry of the molecule shows no usual features. An analysis by *Mogul* (Bruno *et al.*, 2004) using the Cambridge Strucrtural Database (Version 5.32, May, 2012 update; Allen, 2002) reports no |*z*-score| greater than 1 for the bond lengths. The highest |*z*-scores| for bond angles are 1.743 and 1.661 for the C31—C32—C33 and C22—C23—C24 angles, respectively, which are significantly smaller than the 120 ° expected for a benzene ring. This deviation is due to the strained, planar 5-membered C21/C22/C27/C28/C33 ring. The three ring systems of the fluorenyl group is almost planar with an r.m.s. deviation of 0.026 Å of the thirteen atoms from a least squares fitted plane through the atoms. Considering each ring systems individually, central five-membered ring and the two benzene rings of the fluorenyl moiety are all each essentially planar with r.m.s. deviations of 0.0099 Å, 0.0022 Å and 0.0032 Å for the rings C21/C22/C27/C28/C33 (A), C22/C23/C24/C25/C26/C27 (B) and C28/C29/C30/C31/C32/C33 (C) rings, respectively. The dihedral angles between the planes (A) and (B), (A) and (C), and (B) and (C) are 1.34 (9) °, 1.52 (10) °, and 1.51 (7) °, respectively.

In the crystal, molecules are joined into dimeric units by pairs of O—H···O═P hydrogen bonds across crystallographic inversion centers (Fig. 2). The O···O distance at 2.5107 (15) Å is reasonably short indicating moderately-strong hydrogen bonding (Jeffrey, 1997). The dimeric units are joined into continuous chains along the the crystallographic [011] direction by π–π interactions between the fluorenyl ring systems across crystallographic inversion centers. An analysis by *PLATON* (Spek, 2009) shows a ring-to-ring centroid distance of 3.6273 (9)Å for the 5-membered rings (C21/C22/C27/C28/C33) which are co-planar and have a ring slippage distance of 0.825 Å. The perpendicular distances between the benzene rings are 3.4831 (6) and 3.4608 (6) Å.

The packing diagram, Fig. 3, shows columns of phosphinate groups alternating with columns of fluorenyl groups along [100]. The phenyl groups pack parallel to the columns, pointing along [100].

### Experimental

The procedure of Boyd & Regan (1994) was followed. To a solution of phenylphosphinic acid (2.0 g, 14.1 mmol) in dichloromethane (30 ml), diisopropylethylamine (5.16 ml, 29.6 mmol) and trimethylsilyl chloride (3.74 ml, 29.6 mmol) were separately added at 273K under argon. The reaction mixture was stirred at room temperature for 2–3 h, cooled to 273K and 9*H*-9-bromofluorene (3.46 g, 14.1 mmol) was added. After further stirring at room temperature for 3 days, the solvent was removed under vacuum. The residue was suspended in hydrochloric acid (2 *M*, 20 ml) and filtered on a glass frit. The white solid was washed with water and dried giving a yield of 2.30 g (53%) of pure product. IR: 3064 (w), 1592 (*m*), 1476 (w), 1440 (m), 1174 (*vs*), 1131 (*s*), 982 (*vs*), 818 (*s*), 735 (*s*), 717 (*s*), 692 (*s*), 545 (*vs*), 494 (m), 423 (m) cm^-1^. Crystals suitable for single-crystal X-ray analysis were grown from an acetone solution of the title compound in a desiccator with silical gel.

### Refinement

The H atom on O1 was found in the difference Fourier map and its position was allowed to refine freely while its isotropic displacement factor was set to 1.5 times that of O1. The H atoms attached to C atoms were positioned geometrically and allowed to ride on their parent atoms, with C—H bond lengths of 0.95 Å (aromatic CH) and 1.00 Å (*sp*^3^ C), and isotropic displacement parameters equal to 1.2 times *U*_eq_ of the parent atom.

### Crystal data

**Table:**

C_19_H_15_O_2_P	*Z* = 2
*M**_r_* = 306.28	*F*(000) = 320
Triclinic, *P*1	*D*_x_ = 1.346 Mg m^−^^3^
*a* = 8.5736 (7) Å	Mo *K*α radiation, λ = 0.71073 Å
*b* = 9.5668 (8) Å	Cell parameters from 3726 reflections
*c* = 9.6750 (7) Å	θ = 2.2–29.6°
α = 73.348 (5)°	µ = 0.19 mm^−^^1^
β = 87.388 (5)°	*T* = 100 K
γ = 83.619 (6)°	Block, colourless
*V* = 755.49 (10) Å^3^	0.28 × 0.13 × 0.07 mm

### Data collection

**Table:**

Bruker X8 Kappa APEXII diffractometer	4614 independent reflections
Radiation source: sealed ceramic X ray tube, Siemens KFF	3560 reflections with *I* > 2σ(*I*)
Graphite crystal monochromator	*R*_int_ = 0.047
Detector resolution: 8.3333 pixels mm^-1^	θ_max_ = 30.6°, θ_min_ = 3.1°
0.5 ° ω & φ scans	*h* = −12→12
Absorption correction: numerical (*SADABS*; Bruker, 2012)	*k* = −13→13
*T*_min_ = 0.941, *T*_max_ = 0.988	*l* = −13→13
21221 measured reflections	

### Refinement

**Table:**

Refinement on *F*^2^	Primary atom site location: structure-invariant direct methods
Least-squares matrix: full	Secondary atom site location: difference Fourier map
*R*[*F*^2^ > 2σ(*F*^2^)] = 0.044	Hydrogen site location: inferred from neighbouring sites
*wR*(*F*^2^) = 0.112	H atoms treated by a mixture of independent and constrained refinement
*S* = 1.03	*w* = 1/[σ^2^(*F*_o_^2^) + (0.0492*P*)^2^ + 0.2304*P*] where *P* = (*F*_o_^2^ + 2*F*_c_^2^)/3
4614 reflections	(Δ/σ)_max_ = 0.001
202 parameters	Δρ_max_ = 0.33 e Å^−^^3^
0 restraints	Δρ_min_ = −0.41 e Å^−^^3^

### Special details

**Table:**

Experimental. The crystal was cooled to 100 K under a cold nitrogen stream.
Geometry. All e.s.d.'s (except the e.s.d. in the dihedral angle between two l.s. planes) are estimated using the full covariance matrix. The cell e.s.d.'s are taken into account individually in the estimation of e.s.d.'s in distances, angles and torsion angles; correlations between e.s.d.'s in cell parameters are only used when they are defined by crystal symmetry. An approximate (isotropic) treatment of cell e.s.d.'s is used for estimating e.s.d.'s involving l.s. planes.
Refinement. Refinement of *F*^2^ against ALL reflections. The weighted *R*-factor *wR* and goodness of fit *S* are based on *F*^2^, conventional *R*-factors *R* are based on *F*, with *F* set to zero for negative *F*^2^. The threshold expression of *F*^2^ > σ(*F*^2^) is used only for calculating *R*-factors(gt) *etc*. and is not relevant to the choice of reflections for refinement. *R*-factors based on *F*^2^ are statistically about twice as large as those based on *F*, and *R*- factors based on ALL data will be even larger.

### Fractional atomic coordinates and isotropic or equivalent isotropic displacement parameters (Å^2^)

**Table:**

	*x*	*y*	*z*	*U*_iso_*/*U*_eq_	
P1	0.38649 (4)	0.66642 (4)	0.34500 (4)	0.01663 (10)	
O1	0.43045 (13)	0.51673 (11)	0.31276 (11)	0.0229 (2)	
H1	0.468 (2)	0.446 (2)	0.390 (2)	0.034*	
O2	0.46941 (12)	0.68440 (11)	0.47107 (10)	0.0194 (2)	
C11	0.17814 (16)	0.68985 (15)	0.37075 (14)	0.0175 (3)	
C12	0.11582 (17)	0.78050 (16)	0.45427 (15)	0.0211 (3)	
H12	0.1836	0.8302	0.4944	0.025*	
C13	−0.04498 (18)	0.79814 (18)	0.47880 (16)	0.0259 (3)	
H13	−0.0872	0.8602	0.5353	0.031*	
C14	−0.14413 (18)	0.72501 (18)	0.42077 (16)	0.0270 (3)	
H14	−0.254	0.7363	0.4386	0.032*	
C15	−0.08337 (18)	0.63552 (18)	0.33692 (16)	0.0263 (3)	
H15	−0.1517	0.5862	0.297	0.032*	
C16	0.07715 (18)	0.61796 (17)	0.31121 (16)	0.0228 (3)	
H16	0.1185	0.5571	0.2532	0.027*	
C21	0.43723 (16)	0.80119 (15)	0.18013 (14)	0.0169 (3)	
H21	0.5521	0.786	0.1589	0.02*	
C22	0.39388 (16)	0.95586 (15)	0.18998 (14)	0.0177 (3)	
C23	0.44714 (18)	1.02482 (16)	0.28454 (15)	0.0224 (3)	
H23	0.5212	0.9741	0.3566	0.027*	
C24	0.3897 (2)	1.16927 (18)	0.27144 (17)	0.0272 (3)	
H24	0.4249	1.218	0.3353	0.033*	
C25	0.2816 (2)	1.24361 (17)	0.16620 (18)	0.0286 (4)	
H25	0.2444	1.3428	0.1586	0.034*	
C26	0.22646 (19)	1.17511 (17)	0.07137 (17)	0.0251 (3)	
H26	0.1517	1.2262	0.0001	0.03*	
C27	0.28382 (17)	1.02979 (15)	0.08387 (14)	0.0184 (3)	
C28	0.25172 (17)	0.93164 (16)	−0.00072 (14)	0.0190 (3)	
C29	0.15364 (18)	0.95394 (19)	−0.11764 (16)	0.0254 (3)	
H29	0.0904	1.0443	−0.1533	0.031*	
C30	0.15047 (19)	0.8417 (2)	−0.18061 (16)	0.0301 (4)	
H30	0.0847	0.8558	−0.2609	0.036*	
C31	0.2418 (2)	0.7084 (2)	−0.12883 (17)	0.0298 (4)	
H31	0.2379	0.6331	−0.1743	0.036*	
C32	0.33901 (19)	0.68459 (17)	−0.01075 (16)	0.0244 (3)	
H32	0.4007	0.5934	0.0255	0.029*	
C33	0.34340 (17)	0.79710 (16)	0.05227 (14)	0.0183 (3)	

### Atomic displacement parameters (Å^2^)

**Table:**

	*U*^11^	*U*^22^	*U*^33^	*U*^12^	*U*^13^	*U*^23^
P1	0.01661 (18)	0.01688 (17)	0.01648 (17)	−0.00126 (13)	−0.00200 (12)	−0.00478 (13)
O1	0.0300 (6)	0.0182 (5)	0.0207 (5)	0.0024 (4)	−0.0058 (4)	−0.0071 (4)
O2	0.0200 (5)	0.0194 (5)	0.0196 (5)	−0.0018 (4)	−0.0046 (4)	−0.0060 (4)
C11	0.0177 (7)	0.0192 (6)	0.0148 (6)	−0.0048 (5)	−0.0007 (5)	−0.0024 (5)
C12	0.0193 (7)	0.0264 (7)	0.0186 (6)	−0.0042 (6)	−0.0009 (5)	−0.0073 (5)
C13	0.0205 (7)	0.0342 (8)	0.0222 (7)	−0.0016 (6)	0.0021 (6)	−0.0076 (6)
C14	0.0163 (7)	0.0356 (9)	0.0247 (7)	−0.0047 (6)	0.0000 (6)	−0.0009 (6)
C15	0.0224 (8)	0.0301 (8)	0.0256 (7)	−0.0106 (6)	−0.0053 (6)	−0.0031 (6)
C16	0.0243 (8)	0.0234 (7)	0.0216 (7)	−0.0055 (6)	−0.0033 (6)	−0.0062 (6)
C21	0.0151 (6)	0.0191 (6)	0.0169 (6)	−0.0027 (5)	0.0010 (5)	−0.0054 (5)
C22	0.0175 (7)	0.0186 (6)	0.0174 (6)	−0.0052 (5)	0.0049 (5)	−0.0052 (5)
C23	0.0234 (8)	0.0253 (7)	0.0206 (7)	−0.0090 (6)	0.0033 (5)	−0.0078 (6)
C24	0.0330 (9)	0.0273 (8)	0.0269 (8)	−0.0133 (7)	0.0089 (6)	−0.0144 (6)
C25	0.0331 (9)	0.0199 (7)	0.0339 (8)	−0.0058 (6)	0.0124 (7)	−0.0102 (6)
C26	0.0227 (8)	0.0230 (7)	0.0265 (7)	−0.0005 (6)	0.0061 (6)	−0.0037 (6)
C27	0.0186 (7)	0.0199 (7)	0.0164 (6)	−0.0043 (5)	0.0048 (5)	−0.0048 (5)
C28	0.0179 (7)	0.0239 (7)	0.0149 (6)	−0.0043 (5)	0.0032 (5)	−0.0048 (5)
C29	0.0191 (7)	0.0367 (9)	0.0191 (7)	−0.0033 (6)	0.0010 (5)	−0.0057 (6)
C30	0.0247 (8)	0.0501 (10)	0.0190 (7)	−0.0090 (7)	−0.0006 (6)	−0.0135 (7)
C31	0.0322 (9)	0.0407 (9)	0.0244 (7)	−0.0133 (7)	0.0052 (6)	−0.0188 (7)
C32	0.0266 (8)	0.0264 (8)	0.0232 (7)	−0.0066 (6)	0.0061 (6)	−0.0112 (6)
C33	0.0186 (7)	0.0219 (7)	0.0157 (6)	−0.0052 (5)	0.0031 (5)	−0.0066 (5)

### Geometric parameters (Å, º)

**Table:**

P1—O2	1.4997 (10)	C22—C27	1.403 (2)
P1—O1	1.5541 (11)	C23—C24	1.386 (2)
P1—C11	1.7905 (15)	C23—H23	0.95
P1—C21	1.8108 (14)	C24—C25	1.388 (2)
O1—H1	0.90 (2)	C24—H24	0.95
C11—C12	1.396 (2)	C25—C26	1.396 (2)
C11—C16	1.400 (2)	C25—H25	0.95
C12—C13	1.388 (2)	C26—C27	1.395 (2)
C12—H12	0.95	C26—H26	0.95
C13—C14	1.389 (2)	C27—C28	1.4648 (19)
C13—H13	0.95	C28—C29	1.393 (2)
C14—C15	1.387 (2)	C28—C33	1.404 (2)
C14—H14	0.95	C29—C30	1.381 (2)
C15—C16	1.388 (2)	C29—H29	0.95
C15—H15	0.95	C30—C31	1.392 (3)
C16—H16	0.95	C30—H30	0.95
C21—C22	1.5140 (19)	C31—C32	1.395 (2)
C21—C33	1.5181 (19)	C31—H31	0.95
C21—H21	1.0	C32—C33	1.385 (2)
C22—C23	1.3898 (19)	C32—H32	0.95
			
O2—P1—O1	114.59 (6)	C24—C23—C22	118.52 (15)
O2—P1—C11	110.59 (6)	C24—C23—H23	120.7
O1—P1—C11	108.24 (6)	C22—C23—H23	120.7
O2—P1—C21	110.70 (6)	C23—C24—C25	120.83 (15)
O1—P1—C21	104.29 (6)	C23—C24—H24	119.6
C11—P1—C21	108.08 (6)	C25—C24—H24	119.6
P1—O1—H1	113.2 (12)	C24—C25—C26	121.12 (15)
C12—C11—C16	119.49 (13)	C24—C25—H25	119.4
C12—C11—P1	118.86 (11)	C26—C25—H25	119.4
C16—C11—P1	121.64 (11)	C27—C26—C25	118.36 (15)
C13—C12—C11	120.14 (14)	C27—C26—H26	120.8
C13—C12—H12	119.9	C25—C26—H26	120.8
C11—C12—H12	119.9	C26—C27—C22	120.10 (14)
C12—C13—C14	119.98 (15)	C26—C27—C28	130.83 (14)
C12—C13—H13	120.0	C22—C27—C28	109.06 (12)
C14—C13—H13	120.0	C29—C28—C33	120.35 (14)
C15—C14—C13	120.26 (14)	C29—C28—C27	130.97 (14)
C15—C14—H14	119.9	C33—C28—C27	108.67 (12)
C13—C14—H14	119.9	C30—C29—C28	118.50 (15)
C14—C15—C16	120.11 (14)	C30—C29—H29	120.7
C14—C15—H15	119.9	C28—C29—H29	120.7
C16—C15—H15	119.9	C29—C30—C31	121.31 (15)
C15—C16—C11	120.00 (14)	C29—C30—H30	119.3
C15—C16—H16	120.0	C31—C30—H30	119.3
C11—C16—H16	120.0	C30—C31—C32	120.56 (15)
C22—C21—C33	102.97 (11)	C30—C31—H31	119.7
C22—C21—P1	111.40 (9)	C32—C31—H31	119.7
C33—C21—P1	112.47 (9)	C33—C32—C31	118.38 (15)
C22—C21—H21	109.9	C33—C32—H32	120.8
C33—C21—H21	109.9	C31—C32—H32	120.8
P1—C21—H21	109.9	C32—C33—C28	120.90 (14)
C23—C22—C27	121.07 (14)	C32—C33—C21	129.44 (14)
C23—C22—C21	129.34 (13)	C28—C33—C21	109.66 (12)
C27—C22—C21	109.59 (12)		
			
O2—P1—C11—C12	28.54 (13)	C23—C24—C25—C26	0.5 (2)
O1—P1—C11—C12	154.83 (11)	C24—C25—C26—C27	−0.6 (2)
C21—P1—C11—C12	−92.78 (12)	C25—C26—C27—C22	0.1 (2)
O2—P1—C11—C16	−150.46 (11)	C25—C26—C27—C28	−178.32 (13)
O1—P1—C11—C16	−24.18 (13)	C23—C22—C27—C26	0.3 (2)
C21—P1—C11—C16	88.21 (13)	C21—C22—C27—C26	−179.72 (12)
C16—C11—C12—C13	0.5 (2)	C23—C22—C27—C28	179.08 (12)
P1—C11—C12—C13	−178.55 (11)	C21—C22—C27—C28	−0.95 (15)
C11—C12—C13—C14	0.3 (2)	C26—C27—C28—C29	−0.6 (3)
C12—C13—C14—C15	−0.7 (2)	C22—C27—C28—C29	−179.21 (14)
C13—C14—C15—C16	0.4 (2)	C26—C27—C28—C33	178.00 (14)
C14—C15—C16—C11	0.4 (2)	C22—C27—C28—C33	−0.60 (15)
C12—C11—C16—C15	−0.8 (2)	C33—C28—C29—C30	−0.8 (2)
P1—C11—C16—C15	178.15 (11)	C27—C28—C29—C30	177.70 (14)
O2—P1—C21—C22	−59.17 (11)	C28—C29—C30—C31	0.4 (2)
O1—P1—C21—C22	177.10 (9)	C29—C30—C31—C32	0.3 (2)
C11—P1—C21—C22	62.09 (11)	C30—C31—C32—C33	−0.7 (2)
O2—P1—C21—C33	−174.18 (9)	C31—C32—C33—C28	0.3 (2)
O1—P1—C21—C33	62.09 (11)	C31—C32—C33—C21	179.97 (13)
C11—P1—C21—C33	−52.93 (11)	C29—C28—C33—C32	0.4 (2)
C33—C21—C22—C23	−178.06 (13)	C27—C28—C33—C32	−178.41 (12)
P1—C21—C22—C23	61.18 (17)	C29—C28—C33—C21	−179.31 (12)
C33—C21—C22—C27	1.98 (14)	C27—C28—C33—C21	1.90 (15)
P1—C21—C22—C27	−118.78 (11)	C22—C21—C33—C32	177.99 (14)
C27—C22—C23—C24	−0.4 (2)	P1—C21—C33—C32	−61.99 (17)
C21—C22—C23—C24	179.68 (13)	C22—C21—C33—C28	−2.36 (14)
C22—C23—C24—C25	−0.1 (2)	P1—C21—C33—C28	117.67 (11)

### Hydrogen-bond geometry (Å, º)

**Table:**

*D*—H···*A*	*D*—H	H···*A*	*D*···*A*	*D*—H···*A*
O1—H1···O2^i^	0.90 (2)	1.61 (2)	2.5107 (15)	177.9 (19)

Symmetry code: (i) −*x*+1, −*y*+1, −*z*+1.

## References

[bb1] Allen, F. H. (2002). *Acta Cryst.* B**58**, 380–388. 10.1107/s0108768102003890 12037359

[bb2] Boyd, E. A. & Regan, A. C. (1994). *Tetrahedron Lett.* **35**, 4223–4226.

[bb3] Brandenburg, K. (2012). *DIAMOND* Crystal Impact GbR, Bonn, Germany.

[bb4] Bruker (2012). *APEX2*, *SAINT* and *SADABS* Bruker AXS Inc., Madison, Wisconsin, USA.

[bb5] Bruno, I. J., Cole, J. C., Kessler, M., Luo, J., Motherwell, W. D. S., Purkis, L. H., Smith, B. R., Taylor, R., Cooper, R. I., Harris, S. E. & Orpen, A. G. (2004). *J. Chem. Inf. Comput. Sci.* **44**, 2133–2144. 10.1021/ci049780b 15554684

[bb6] Burrow, R. A., Farrar, D. H., Lough, A. J., Siqueira, M. R. & Squizani, F. (2000). *Acta Cryst.* C**56**, e357–e358.

[bb7] Burrow, R. A. & Siqueira da Silva, R. M. (2011*a*). *Acta Cryst.* E**67**, o1045. 10.1107/S1600536811008245 PMC3089092 21754372

[bb8] Burrow, R. A. & Siqueira da Silva, R. M. (2011*b*). *Acta Cryst.* E**67**, o2005. 10.1107/S1600536811025530 PMC3213460 22091039

[bb10] Burrow, R. A. & da Silva, R. M. S. da (2012). *Acta Cryst.* E**68**, o3488. 10.1107/S160053681204812X PMC3589060 23476296

[bb11] Jeffrey, G. A. (1997). *An Introduction to Hydrogen Bonding*, pp. 11–16. New York: Oxford University Press.

[bb12] Sheldrick, G. M. (2008). *Acta Cryst.* A**64**, 112–122. 10.1107/S0108767307043930 18156677

[bb13] Siqueira, M. R., Tonetto, T. C., Rizzatti, M. R., Lang, E. S., Ellena, J. & Burrow, R. A. (2006). *Inorg. Chem. Commun.* **9**, 536–540.

[bb14] Spek, A. L. (2009). *Acta Cryst.* D**65**, 148–155. 10.1107/S090744490804362X PMC2631630 19171970

[bb15] Vioux, A., Le Bideau, J., Hubert Martin, P. & Leclerq, D. (2004). *Top. Curr. Chem.* **232**, 145–174.

[bb16] Westrip, S. P. (2010). *J. Appl. Cryst.* **43**, 920–925.

